# Effect of recombinant human brain natriuretic peptide (rhBNP) versus nitroglycerin in patients with heart failure: A systematic review and meta-analysis

**DOI:** 10.1097/MD.0000000000004757

**Published:** 2016-11-04

**Authors:** Sijie Zhang, Zhiqian Wang

**Affiliations:** a Department of Cardiology, The Fourth Hospital of Hebei Medical University; b Department of Cardiology, The Third Hospital of Hebei Medical University, Shijiazhuang, Hebei, P.R. China.

**Keywords:** heart failure, hypotension, mortality, readmission, renal dysfunction, rhBNP

## Abstract

**Background:**

This study was the first to evaluate the therapeutic outcomes of recombinant human brain natriuretic peptide (rhBNP) versus nitroglycerin (NIT) in patients with heart failure (HF).

**Methods:**

The electronic databases were systematically searched to identify available studies. The pooled odds ratios (ORs) and their 95% confidence intervals (95% CIs) were analyzed to assess the mortality, readmission, hypotension, and renal dysfunction in the comparison of rhBNP and NIT therapies.

**Results:**

Final 5 randomized controlled trials (RCTs) involving 782 patients with HF were carried out in our study. The pooled OR of mortality, readmission, and hypotension showed that no significant difference was found in both drugs (*P* > 0.05), with the absence of heterogeneity. The incidence of renal dysfunction was not significant difference in both groups (*P* = 0.85). The pooled OR from 2 studies of Asian population using multivariate analysis demonstrated that the use of rhBNP was correlated with a significantly decreased risk of renal dysfunction (*I*^2^ = 0.0%, OR = 0.19, *P* = 0.001). Possible publication bias was not detected using Egger's test (*P* > 0.05).

**Conclusions:**

The results suggested that rhBNP and NIT therapies were not significant difference in mortality, readmission, and hypotension. The use of rhBNP may become a useful predictor of renal dysfunction in Asian patients with HF. Additional studies are needed for Caucasian population with HF.

## 1 Introduction

Heart failure is a complex syndrome of cardiac dysfunction, and the late common outcome of many heart diseases.^[1]^ HF has become a growing global public health problem with an estimated prevalence of > 41 million patients in 2010, especially higher prevalence rates are observed after the age of 65 years.^[2]^ Moreover, HF causes a rising burden with approximately $108 billion in drug costs annually through the world.^[3]^ HF has been found to be linked with many risk factors, including hypertension, obesity, diabetes, myocardial related diseases, biomass smoke exposure, sedentary lifestyle, stress, dyslipidemia, and so on.^[4,5]^ HF is not easily and accurately diagnosed because of the absence of the organ-specific signs and symptoms.^[6]^ Despite advancements in medical treatment management and patient care, the 5-year mortality rate of HF has been estimated as about 50% to 60%.^[7,8]^

For many years, there have been major improvements in the therapeutic options of HF, many pharmacologic therapies have been evaluated to gain the clinical practice guidelines in clinical trials, such as nesiritide, diuretics, nitrates, inotropes, nitroglycerin, nopamine, hypertonic saline, and so on.^[9,10]^ Nesiritide, rhBNP, approved by the Food and Drug Administration (FDA) for the therapy of acute decompensated heart failure (ADHF) in 2001.^[11]^ rhBNP has multiple functions, including facilitating natriuresis, diuresis, inhibiting renin–angiotensin–aldosterone system (RAAS), increasing cardiac output, decreasing pulmonary capillary wedge pressure (PCWP) and improving diastolic function.^[12–15]^ In addition, NIT is also an effective treatment drug for assisting with the management of HF patients through the final reduction of cardiac filling pressures and an increase of CO.^[10]^ Several studies have reported the potent therapeutic effects of rhBNP in patients with HF.^[16,17]^

However, a systematic comparison between rhBNP and NIT in the treatment of HF patients remains unclear. The current study was the first to investigate the effect of rhBNP versus NIT on mortality rate, readmission rate, hypotension, and renal dysfunction in patients with HF.

## 2 Materials and methods

### 2.1 Literature search

We carefully searched a range of digital databases (PubMed, EBSCO, Cochrane Library, and ScienceDirect) to identify potential articles published in English up to June 26th, 2016. The relevant key words and text word strategy were applied: “Brain Natriuretic Peptide OR Recombinant human brain natriuretic peptide OR Nesiritide OR Natrecor OR B-Type Natriuretic Peptide,” “Nitroglycerin OR Nitrol OR Nitroglyn OR Nitrostat OR Glyceryl Trinitrate OR Gilustenon,” “Heart Failure OR Cardiac Failure OR Myocardial Failure.” Moreover, we also checked the references of the articles identified to get more relevant studies.

### 2.2 Selection criteria

The eligible publications were determined if they satisfied the following inclusion criteria: (1) articles published in English using human samples were included in the study; (2) high-quality studies were RCTs; (3) studies were compared rhBNP with NIT; (4) patients had to be confirmed for the diagnosis of HF, and include physical examination and appropriate laboratory tests, and so on;^[18]^ (5) studies had to provided original data with sufficient information to evaluate the effect of rhBNP and NIT; (6) study with the latest or most complete data was selected when >1 article using the same samples was published.

### 2.3 Ethics committee and consent to participate

The current study was not a primary research involving humans or animals but was a secondary analysis of human sample data available in the public domain.

### 2.4 Data extraction

For eligible studies, the following data were extracted based on inclusion criteria: surname of the first author, year of publication, country, ethnicity, trial design, sample sizes, follow-up time, levels of NIT and rhBNP, mortality, re-admission, renal dysfunction, and hypotension. Disagreements were discussed by the authors. Data from each article were independently collected by 2 authors.

### 2.5 Statistical analysis

All data analyses were carried out using Stata version 12.0 software (STATA Corp., College Station, TX) in this study. The odds ratios (ORs) and their 95% confidence intervals (95% CIs) from the original article providing multivariate analysis results were calculated to determine whether rhBNP treatment was an independent predictor of renal dysfunction in HF. The pooled ORs with corresponding 95% CIs were also calculated to assess the outcomes of rhBNP and NIT drugs in patients with HF. The heterogeneity of the studies was examined using the chi-square test.^[19]^ The overall OR value was calculated and summarized under the random-effects model. Egger's test was applied to evaluate the potential publication bias.^[20]^ A *P* value of <0.05 was considered be statistically significant.

## 3 Results

### 3.1 Study characteristics

**Figure 1:**
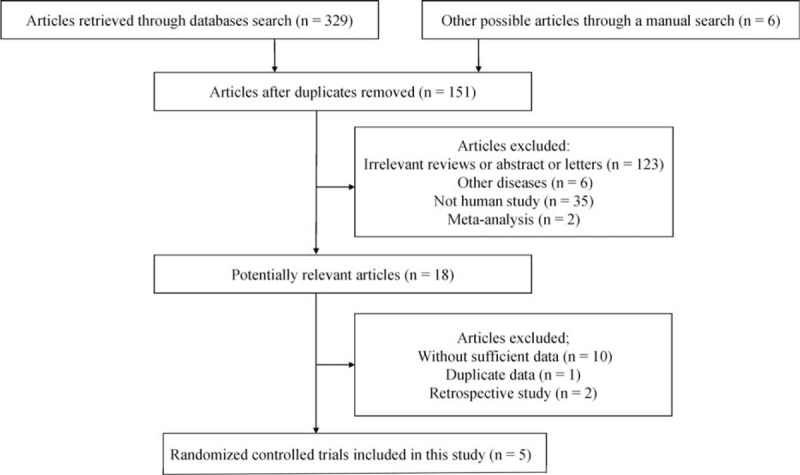
Flow diagram of the literature search strategy in this study.

A total of 335 potentially relevant articles were retrieved from the initial search. According to the above inclusion criteria, as shown in Fig. 1, final analyses of 5 RTCs involving 782 patients with HF were performed in this meta-analysis,^[14,21–24]^ including rhBNP (n = 419) and NIT group (n = 363). Five RCTs had a range of 1 to 6-month follow-up. In addition, 2 studies were performed in China^[21,22]^ and the remaining 3 studies were conducted in the USA.^[14,23,24]^ An RCT with >2 scores was considered to be high quality based on the Jadad scale.^[25]^ In total 5 eligible studies met a score ≥3. The major characteristics of the included studies were shown in Table 1.

**Table 1:**
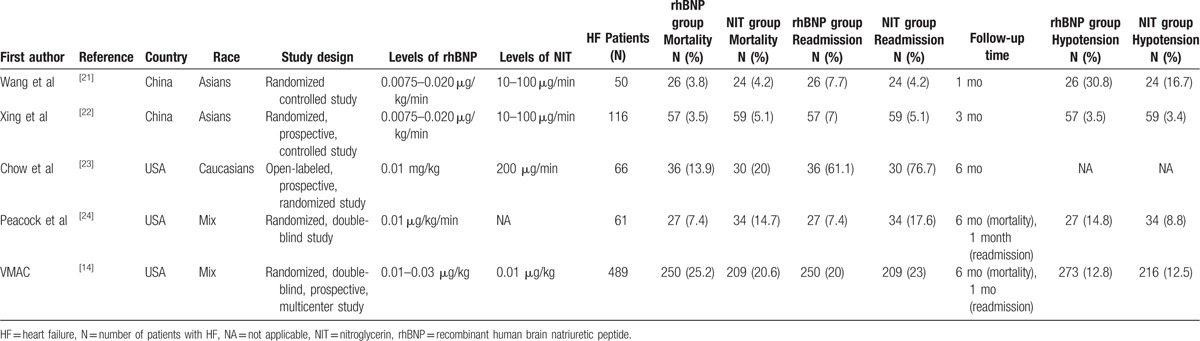
The main characteristics of the included studies.

### 3.2 Analysis of mortality rate

**Figure 2:**
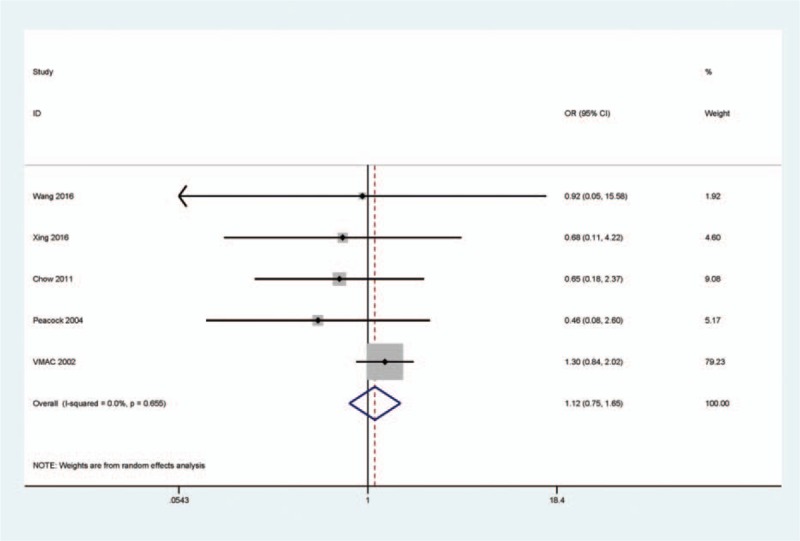
Forest plot indicating the pooled OR from 5 studies with 396 rhBNP and 356 NIT patients for mortality rate in rhBNP vs NIT treatments in HF patients, *I*^2^ = 0.0%, OR = 1.12, 95% CI = 0.75–1.65, *P* = 0.585. HF = heart failure, NIT = nitroglycerin, rhBNP = recombinant human brain natriuretic peptide.

There was no evidence of a heterogeneity in the mortality rate (*I*^2^ = 0.0%). Compared with the NIT group, as shown in Fig. 2, the pooled OR from 5 studies with 396 rhBNP and 356 NIT patients demonstrated that rhBNP and NIT groups had a similar mortality rate (OR = 1.12, 95% CI = 0.75–1.65, *P* = 0.585), suggesting that rhBNP therapy was not correlated with a risk of mortality.

### 3.3 Analysis of readmission rate

**Figure 3:**
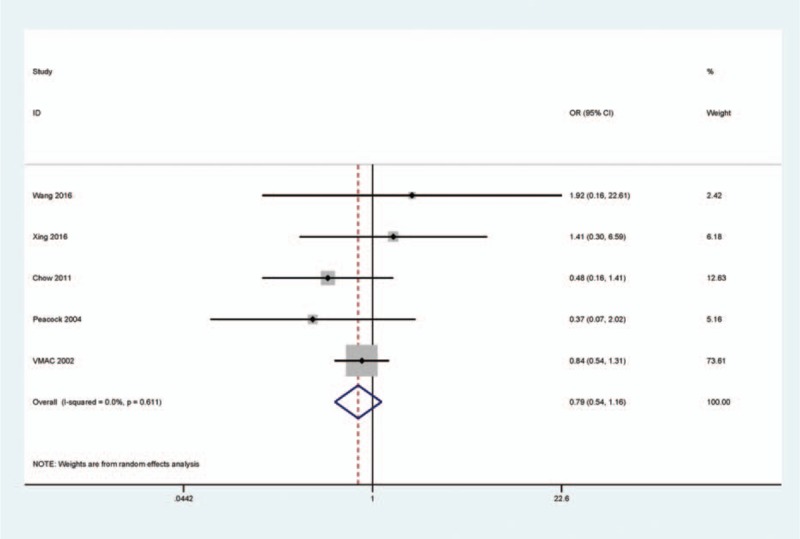
Forest plot indicating the pooled OR from 5 studies with 396 rhBNP and 356 NIT patients for readmission rate in rhBNP vs NIT treatments in HF patients, *I*^2^ = 0.0%, OR = 0.79, 95% CI = 0.54–1.16, *P* = 0.226. HF = heart failure, NIT = nitroglycerin, rhBNP = recombinant human brain natriuretic peptide.

When the rhBNP group (n = 396) was compared to the NIT group (n = 356), the overall OR from 5 studies revealed that the readmission rate had a similar OR value in rhBNP and NIT groups (Fig. 3), suggesting that rhBNP therapy was not correlated with a risk of readmission (*I*^2^ = 0.0%, OR = 0.79, 95% CI = 0.54–1.16, *P* = 0.226).

### 3.4 Analysis of hypotension

**Figure 4:**
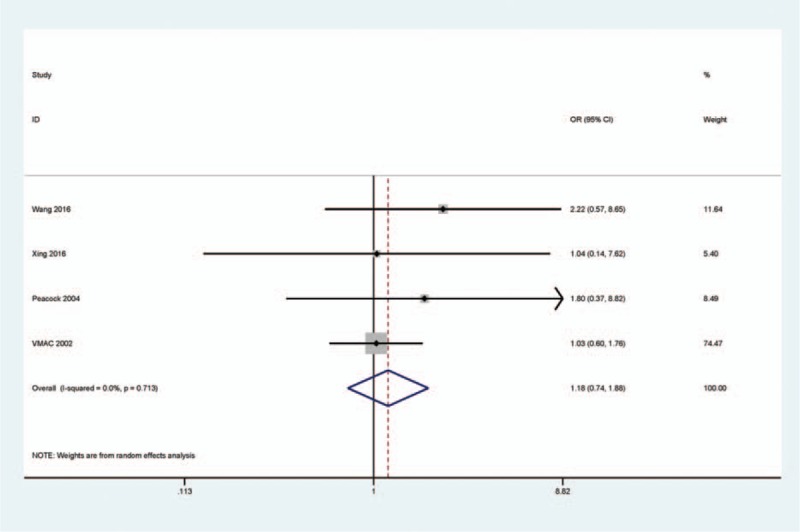
Forest plot indicating the pooled OR from 4 studies with 383 rhBNP and 333 NIT patients for hypotension in rhBNP vs NIT treatments in HF patients, *I*^2^ = 0.0%, OR = 1.18, 95% CI = 0.74–1.88, *P* = 0.482. HF = heart failure, NIT = nitroglycerin, rhBNP = recombinant human brain natriuretic peptide.

The pooled OR from 4 studies involving 383 rhBNP and 333 NIT patients showed that the OR of rhBNP and NIT groups was similar in hypotension (Fig. 4), which suggested that rhBNP therapy was not correlated with a risk of hypotension (*I*^2^ = 0.0%, OR = 1.18, 95% CI = 0.74–1.88, *P* = 0.482).

### 3.5 Analysis of renal dysfunction

As shown in Table 2, in the comparison of rhBNP and NIT therapies, a substantial heterogeneity was observed in renal dysfunction (*I*^2^ = 88.1%). The outcome showed that rhBNP and NIT groups had a similar OR in renal dysfunction (OR = 1.31, 95% CI = 0.08–21.03, *P* = 0.85), including 2 studies with 67 rhBNP and 64 NIT patients, which indicated that rhBNP treatment was not associated with a risk of renal dysfunction. We further extracted the initial data using multiple logistic regression analysis to assess whether rhBNP treatment was correlated with a risk of renal dysfunction. The result of multiple logistic regression from 2 studies involving 166 Asian patients with HF showed that the use of rhBNP was significantly associated with a decreased risk of renal dysfunction (*I*^2^ = 0.0%, OR = 0.19, 95% CI = 0.07–0.50, *P* = 0.001).

**Table 2:**
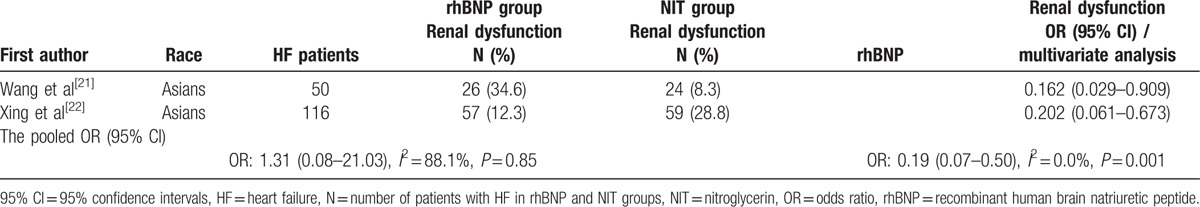
Analysis of renal dysfunction in patients with HF.

### 3.6 Publication bias

**Figure 5:**
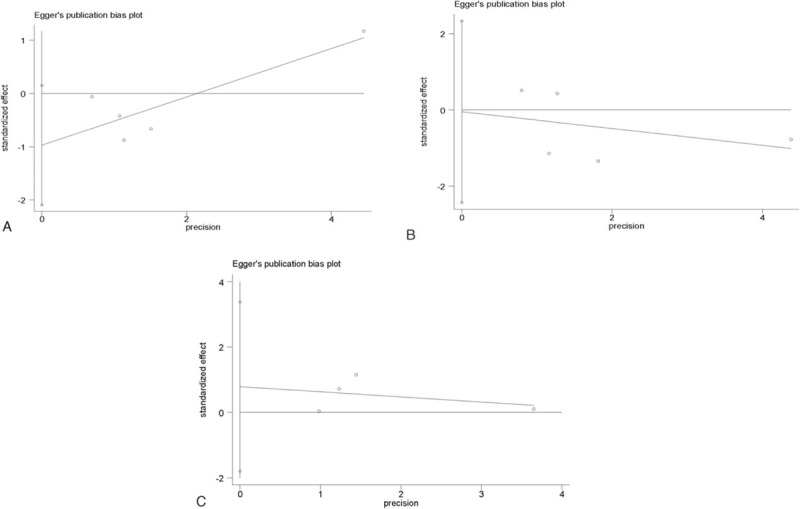
Funnel plot of publication based on the Egger linear regression test in rhBNP vs NIT treatments in HF patients. (A) Mortality rate (*P* = 0.07); (B) readmission rate (*P* = 0.956); (C) hypotension (*P* = 0.32). HF = heart failure, NIT = nitroglycerin, rhBNP = Recombinant human brain natriuretic peptide.

The possible publication bias was determined using the Egger linear regression test (Fig. 5). The result demonstrated that no significant publication bias was found in this study (all *P* > 0.05), suggesting that our analysis was stable and reliable.

## 4 Discussion

Mainly depending on severity of symptoms, heart dysfunction, patient age, and other factors, HF is frequent hospitalization diagnosis correlated with high mortality and readmission rates.^[26,27]^ Some studies have shown that rehospitalizations are important health outcomes for patients with HF and serve as useful health-care utilization.^[28,29]^ Thus, there is a need for HF patients to reduce hospital admissions, mortality, and relieve symptoms. The current study of rhBNP and NIT therapies evaluated 5 RCTs, with a range of 1 to 6-month follow-up. However, there were inconsistent results. The use of rhBNP had different mortality rate, with a range of 3.5% to 25.2%.^[14,22]^ NIT treatment had also different mortality rate, with a range of 4.2% to 20.6%.^[14,21]^ Readmission rate ranged from 7% to 61.1% in the rhBNP group.^[22,23]^ Readmission rate ranged from 4.2% to 76.7% in the NIT group.^[21,23]^ The highest readmission rate belonged to the result of 6-month follow-up. Compared with NIT, our findings from 5 studies with 396 rhBNP and 356 NIT patients demonstrated that rhBNP and NIT groups were not significantly different in mortality and readmission (*P* > 0.1), suggesting that rhBNP neither increased nor decreased the risk of mortality and readmission. Possible publication bias was not observed in our study, indicating the stability of the results.

In addition, the adverse events of rhBNP therapy in patents with HF include hypotension, headache, nausea, decreased heart rate, and renal dysfunction.^[24,30]^ Similarly, the side effects of NIT treatment were also commonly observed in hypotension and renal dysfunction.^[14,21]^ Some studies show that rhBNP treatment may increase a risk of renal dysfunction and the main contribution might be hypotension.^[30–32]^ The current study of hypotension from 4 studies involving 383 rhBNP and 333 NIT patients showed that no significant difference was found in both groups (*P* = 0.482). In addition, no evidence of publication bias was observed, suggesting the stability of the analysis. The incidence of renal dysfunction from 2 studies demonstrated that rhBNP and NIT treatments had no significant difference. However, a substantial heterogeneity was found (*I*^2^ = 88.1%), suggesting that the result was inconsistent. Thus, additional studies with larger sample size are essential to achieve the consistent conclusion on the incidence of renal dysfunction in the future. Next, we extracted the original results of multiple logistic regression analysis to determine whether rhBNP was associated with a risk of renal dysfunction. Our result revealed that rhBNP treatment was significantly correlated with a decreased risk of renal dysfunction (OR = 0.19, *P* = 0.001), suggesting that the use of rhBNP may be an independent predictor of renal dysfunction in Asian patients with HF. However, the result of renal dysfunction should be cautious as only 2 studies with small subjects were included in our study.

Several limitations of this study should be carefully considered. First, although we searched papers as completely as possible based on the above electronic databases, only articles published in English were included in the present study, other papers published in other language and other styles such as conferences abstract were missed, which may cause a selection bias. Second, the total sample subjects involving 5 RCTs were not sufficient larger (< 1000)^[33]^; our results may lack vigorous power on the analyses of mortality, readmission, hypotension, and renal dysfunction in hrBNP vs NIT. Thus, more well-designed studies with lager sample size are very essential to further validate our study in the future. Third, the data of renal dysfunction using multivariate analysis were lacking in Caucasian population; the following study is a need to assess whether the use of rhBNP is also a predictive factor of renal dysfunction for Caucasian patients with HF.

In conclusion, when rhBNP was compared with NIT in HF patients, our study suggested that the results of both groups were not significantly different in mortality, readmission, and hypotension. Interestingly, the use of rhBNP was an independent predictor of renal dysfunction in Asian patients with HF. More studies comprising larger sample sizes are essential to further confirm our results in the future, especially an analysis of renal dysfunction based on multiple logistic regression for Caucasian patients with HF.
